# Should Workhouse Matrons Be Trained Nurses?

**Published:** 1913-08-16

**Authors:** J. Wilson

**Affiliations:** Hon. Treasurer Workhouse Nursing Association.


### EDITOR'S LETTER-BOX.

### Should Workhouse Matrons be Trained Nurses?

*To the Editor of* The Hospital.

Sir,—You invite correspondence on the question of *always* "appointing a trained nurse as matron in workhouses." May I offer a few views founded on a fairly wide experience of country Unions? First, I will take the case for such appointments and their advantages and drawbacks. (1) Guardians prefer a married couple, and I think there is no Order prescribing that a master should marry a trained nurse! Therefore we would have, as a rule, single women applying as matrons; the master might be single also, or married, but the reform of work at the very slow stage at which we are proceeding would, in many cases, be causes of friction of various kinds. I shall be glad to hear of cases where such a plan has turned out really well, because the proposition has been little tried so far as we have had experience. I know *one* case where it has answered admirably in a tiny Union. (2) There are great advantages, though I admit drawbacks, in a master and matron being fully in touch in many, if not all, points of their work, and here we come upon a difficulty. Trained nurses are not generally trained except in nursing; they can *learn* to manage the work of managing workhouse sick wards, dietetics, etc., but how are they to obtain experience in the very varied duties essential to a good workhouse matron? These are more exacting than most imagine, and require a great deal of time:—supervision of the infants and some of the able bodied, visitation of tramps—women; the care of the laundry, and often cutting out clothing, the employment of the able-bodied women, who are often semi-imbecile, represent only some of the workhouse matron's duties. I think the matron's time is thus fully employed, especially as she often has one or two children of her own who require some attention in all conscience. I have found the matron cooking the dinner for the whole "House"; another helping in the laundry when labour was "scarce." How can she have time to even supervise the care of the sick except in a *small* Union?—in these I consider necessary nursing knowledge is essential, as nurses leave constantly, while the matron has, as a rule, husband and children and promotion to consider. (3) Nurses are in great demand; as you say, they will not willingly take work in old-fashioned Unions. In our Association nurses have stayed on, deeply pitying the sick; their health has been completely ruined by doing so, although these admirable women have retired on pension, they are almost all subjects of heart disease or rheumatism, often caused by the state of old Unions which the Local Government Board had desired to be abandoned, or by overwork. These Unions are not abandoned or improved yet in some cases; in Ireland, if an Order is desirable and the Board of Guardians recalcitrant, power is given to the Irish Local Government Board to disband the Guardians, to send two officials to take their place and thus to bring matters up to date as far as possible. This is required in England, because your columns show weekly how badly, how ignorantly power is used by many Boards—ignorance plays a large part in the cases we all know. My opinion is, and I have visited over 250 country Unions, that the great essential is specialisation—namely, to leave the master and matron to their own very important work, but to revise all rules for the nurse or superintendent nurse. Give her, as in a cottage hospital, *responsibility*, allow her to call in the doctor when required, relieve master and matron of responsibility for the sick wards, and let all responsibility fall on her shoulders as accountable to the Guardians. Then there will be less gossip and bitterness; the nurse will know she must both act and think wisely. She should not be placed, as now, between two fires. Really her work is hard enough if well done.—Yours truly,

J. Wilson,
Hon. Treasurer Workhouse Nursing Association.

August 9, 1913.

